# Bacteriophage PAK_P3 genome structuration and dynamics during infection of *Pseudomonas aeruginosa* reveal specific interaction patterns

**DOI:** 10.1126/sciadv.aef2512

**Published:** 2026-08-26

**Authors:** Amaury Bignaud, Quentin Lamy-Besnier, Devon E. Conti, Agnès Thierry, Fabien Girard, Pauline Misson, Romain Koszul, Laurent Debarbieux, Martial Marbouty

**Affiliations:** ^1^ Institut Pasteur, Université Paris Cité, Spatial Regulation of Genomes Group, CNRS UMR 3525, Paris F-75015 France.; ^2^ Sorbonne Université, Collège Doctoral, Paris, France.; ^3^ Institut Pasteur, Université Paris Cité, CNRS UMR6047, Bacteriophage Bacterium Host, Paris F-75015, France.; ^4^ Department of Biochemistry, University of Oxford, Oxford, UK.

## Abstract

While the dynamic changes in genome organization in cellular organisms have been well described, the three-dimensional (3D) folding of phage genomes during the infection of their host is extremely limited. Understanding how phage genomes fold, invade, and rearrange the genome of their host to functionally organize the optimal expression of their genes remains unknown. Here, we explore the spatial dynamics of the virulent double-stranded DNA phage PAK_P3 during infection of its host *Pseudomonas aeruginosa* and reveal how its genome rapidly decondenses to adopt a specific 3D organization that reflects its transcriptional program. Concomitantly, the host genome decondenses as gene expression wanes and transcription-induced domains vanish. We also uncover specific contacts of the phage genome with host chromosome, showing how the phage genome exploits the spatial genome architecture of its host to succeed in its infection cycle. Our data highlight an unprecedented level of genome folding and gene expression regulation during a viral infection.

## INTRODUCTION

Mobile genetic elements (MGEs) are ubiquitous and play a major role in shaping the structure, evolution, and function of genomes across all domains of life (*1*, *2*). These elements—including transposons, plasmids, integrons, retroelements, and viruses—are often called genomic “parasites” as they can replicate and move within and between host genomes. Their mobility affects genome organization (*3*–*5*) and facilitates horizontal gene transfer driving rapid evolution (*6*). While genetic and evolutionary consequences of MGEs have been well studied, how MGEs organize themselves into three dimensions and how they influence the physical and spatial organization of host genomes remain understudied.

The advent of proximity ligation technologies [high-throughput chromosome conformation capture (HiC)] has transformed our understanding of genome biology (*7*, *8*). These techniques allow to capture how genomes fold into three dimensions inside cells, revealing how chromosomes are organized and how spatial proximity relates to essential processes such as transcription, replication, or DNA repair. In eukaryotes, these studies have uncovered principles such as chromatin looping and compartmentalization (*8*–*11*). In bacteria, HiC has revealed robust structural features such as self-interacting domains [chromosome interaction domains (CIDs)] (*12*), alignment of chromosome arms (*13*, *14*), links between gene expression and chromatin contacts (*15*, *16*), or trans interactions between chromosomes to synchronize their replication (*17*). Nevertheless, few studies have investigated the three-dimensional (3D) relationships between MGEs and host chromosomes using model organisms (*18*–*22*). For instance, DNA of the hepatitis B virus associates closely with CpG islands located upstream of active genes in infected human cells, presumably favoring their own replication and gene expression through proximity with transcriptional machinery (*19*). In contrast, yeast *Saccharomyces cerevisiae* 2-μ plasmid appears to tether preferentially, but loosely, to regions with low transcriptional activity, potentially to facilitate its hitchhiking segregation strategy (*18*). These few examples suggest that MGEs exploit or adapt to the spatial architecture of host genomes to orchestrate their life cycle.

Among MGEs, virulent phages stand out as they inject their genome into their bacterial host to hijack its resources and produce new viral particles that are released upon cell lysis, a process that can be achieved in less than 10 min (*23*). While the transcriptional and biochemical processes taking place during phage infection have been described for many diverse phages (*24*–*26*), little is known regarding the structural transitions of phage genomes themselves, from inside the capsid to delivery into the host and during infection cycle. Today, the question of whether the genome of virulent phages exploits the spatial architecture of their host to favor their infection strategy or has selected an independent mechanism remains open. Similarly, how phage infection influences the spatial genome organization of its host is still unknown.

To explore these questions, we combined time-series HiC with transcriptomics to investigate the genome folding dynamics of the virulent phage PAK_P3 during infection of the opportunistic pathogen *Pseudomonas aeruginosa*. This phage [∼88-kb linear double-stranded DNA (dsDNA)] belongs to the *Nankokuvirus* family and was shown to hijack the host RNA polymerase for its own transcription (*26*, *27*). With an eclipse period of nearly 12 min and an infection cycle duration of around 20 min, phage PAK_P3 is among one of the fastest known dsDNA phages with contractile tails (*27*). By tracking in parallel the variation of phage and bacterial genome architecture as well as their interactions during infection kinetics, we revealed (i) an overall decondensation of the *P. aeruginosa* chromosome, (ii) a rapid and specific dynamics of PAK_P3 genome folding linked to its transcriptional program, and (iii) a specific interaction pattern between both genomes, pointing to the existence of dedicated mechanisms set up by the phage to better exploit its host’s cellular machinery and/or interfere with the host’s protective response.

By leveraging spatial genomics applied to phage biology, this work sheds light on the mechanisms of phage genome expression, their interactions with hosts, and their subversion mechanisms. The overall results deepen our understanding of phage-host relationships.

## RESULTS

### High-resolution chromosome architecture of *P. aeruginosa* strain PAK

**Fig. 1.**
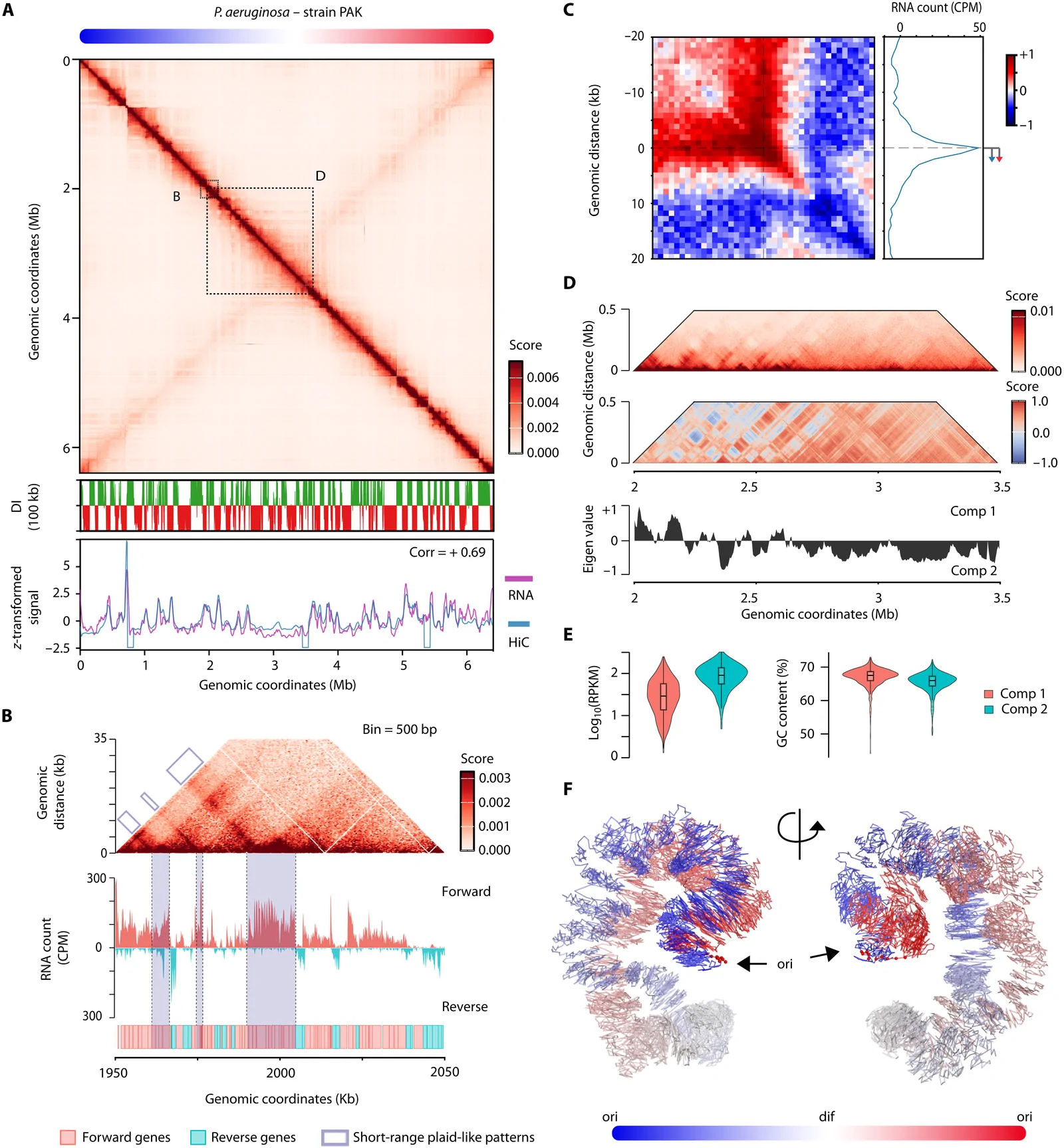
*P. aeruginosa* strain PAK chromosome folding. (**A**) From top to bottom: 5-kb-resolution HiC contact maps of the strain PAK; directional index (DI) at 100 kb that measures the bias in contact frequency upstream and downstream of an HiC region revealing CIDs; correlation between the DNA short-range contacts (blue, 5-kb bins and 25-kb distance) and the transcriptional signal (purple, 5-kb bin) (Pearson correlation score = +0.69). A linear scheme of the chromosome with a colored gradient (blue-white-red) is indicated above the contact map and refers to the average 3D structure in (E). Dashed squares indicate zoom area for (B) and (D), respectively. (**B**) Magnification of a genomic locus at 500-bp resolution (1.95 to 2.05 Mb). Below the contact map is plotted the RNA signal in counts per million (CPM) for the forward (red) and reverse (blue) (bin = 100 bp). The scheme below the plot indicates the orientation of the genes (red, forward; blue, reverse). Blue square indicates examples of compartmented highly transcribed areas (i.e., TIDs and plaid-like patterns). (**C**) Pileup log_10_ ratio of 20-kb contact map windows (bin = 1 kb) centered on the start codons of the 10% most transcribed transcription units compared to random windows taken along the main diagonal. On the right is the corresponding pileup of transcription tracks (red arrow, forward genes; blue arrow, reverse genes). (**D**) Zoom of a larger part of the contact map (2 to 3.5 Mb) using either balanced HiC (top) or autocorrelation scores (bottom) (bin = 5 kb). Eigen vector 3 value highlighting the existence of two compartments (comp 1 and comp 2). (**E**) Violin plot of the distribution of transcription signal and GC content within 5-kb bins in the two detected compartments (red, comp 1; blue, comp 2). (**F**) Average 3D projection of the PAK chromosome. Colors along the structure correspond to the colors of the linear scheme of the chromosome. Origin of replication (ori) corresponds to larger red beads and is indicated.

To establish a robust baseline for assessing phage infection–induced chromosomal changes, we first characterized the 3D organization of the genome of *P. aeruginosa* strain PAK (host of the phage PAK_P3) from an exponential culture using a HiC protocol optimized for bacteria. We identified ubiquitous folding features previously reported for model bacteria such as *Escherichia coli*, *Bacillus subtilis*, as well as *P. aeruginosa* strain PAO1 (*14*, *15*, *28*, *29*): (i) a prominent diagonal of strong local contacts, indicative of physical proximity between neighboring loci (Fig. 1A and fig. S1); (ii) large self-interacting regions dubbed CIDs (*n* = 37) whose boundaries correspond to highly transcribed genes (Fig. 1A); and (iii) a general correlation between gene expression, as assessed from published RNA sequencing experiments (*27*), and short-range contacts (Pearson correlation score = +0.69; Fig. 1A) (*15*, *16*). High-resolution [500 base pairs (bp)] contact maps highlighted the fine-scale bacterial gene structure, with transcription units enriched in short-range HiC contacts and forming transcription-induced domains (TIDs) that interact preferentially with each other (Fig. 1B, blue squares), as recently described in *Vibrio cholerae* and *E. coli* (*16*). Pileup analysis of averaged contact maps centered on the start codons of the 10% most transcribed transcription units further revealed the same bundle signature at the transcription start sites as previously reported (Fig. 1C) (*16*). We also detected a plaid-like pattern at larger scales and visible across the entire contact map, especially when we plotted the autocorrelation score of part of the matrix (Fig. 1, A and D). This pattern, reminiscent of the alternation between transcriptionally active and inactive domains common in eukaryotic genomes (*8*), demonstrates the existence of compartment-like in bacteria (compartments 1 and 2) and was detectable using the third eigen vector of the matrix (Fig. 1D, bottom part, and fig. S2). Analysis of transcription signal inside these two compartments shows a clear enrichment of transcription activity in the second compartment (Fig. 1E). Last, *P. aeruginosa* contact map exhibited a characteristic diagonal perpendicular to the main one, extending from the *parS* cluster near the origin of replication, toward the terminus, reflecting long-range contacts between chromosome replichores, a pattern mediated by SMC-ScpAB complexes (Structural and Maintenance of Chromosomes) (Fig. 1A and fig. S1A) (*13*, *14*, *28*, *30*). Average 3D projection of the contact map, generated using a probability-based approach based on negative binomial random variables (*31*–*33*), recapitulated the global architecture of *P. aeruginosa* chromosome and the cohesion between the two chromosomal replichores (Fig. 1F). These observations confirmed that HiC captures global and local features of chromosome architecture and was, therefore, suitable to detect potential minimal structural transitions during phage infection.

### PAK_P3 infection leads to a local destructuration of the host chromosome

**Fig. 2.**
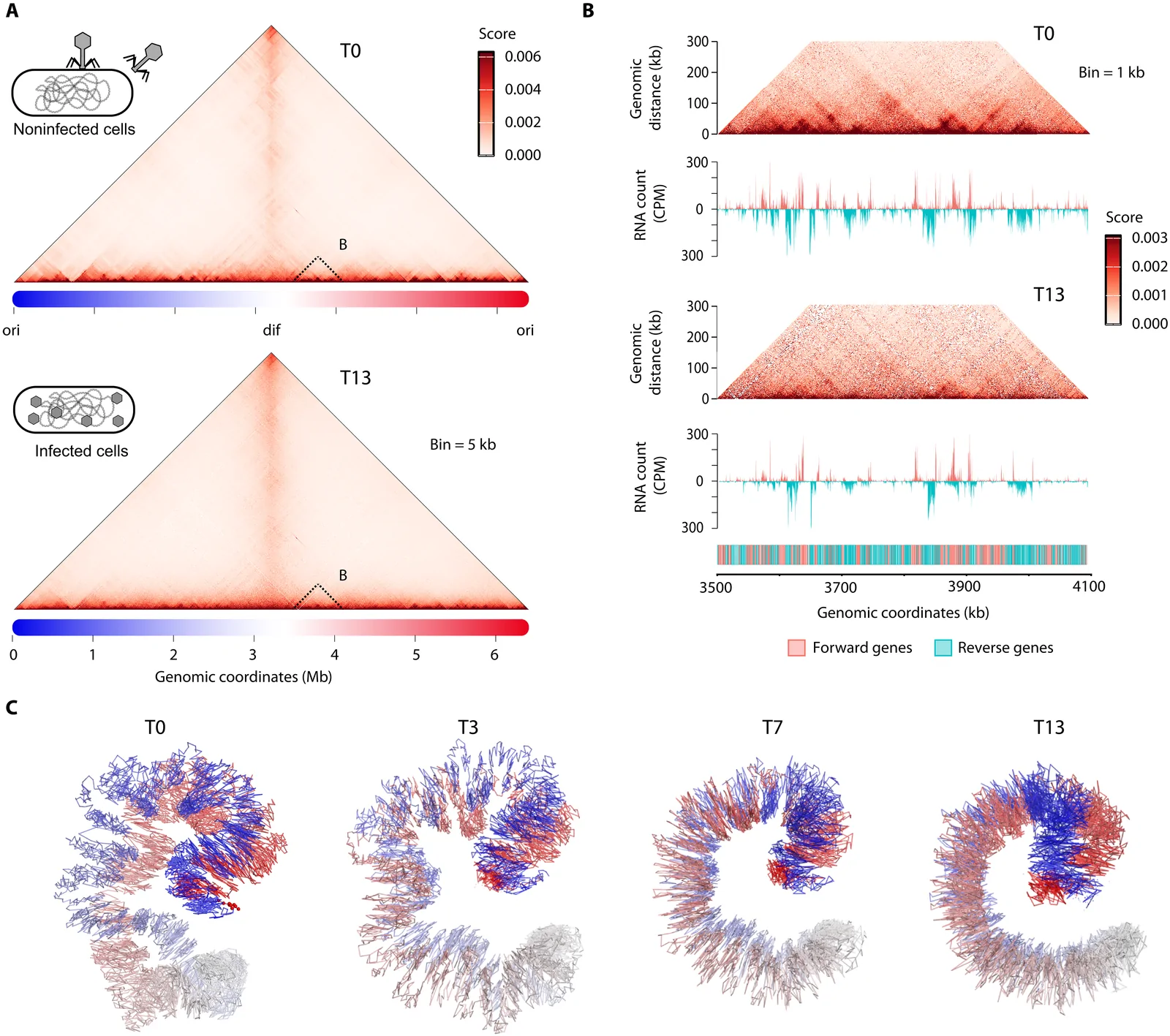
Impact of phage PAK_P3 infection on *P. aeruginosa* chromosome architecture. (**A**) Contact maps at 5-kb resolution at T0 (top) and T13 (bottom). A linear scheme of the chromosome with a colored gradient (blue-white-red) is indicated below the contact maps and refers to the average 3D structure in (C). (**B**) Magnification of a genomic locus (3.5 to 4.1 Mb) at T0 and T13 revealing the impact of phages on genome architecture at short scale (bin = 1 kb). Below the two contact maps, the RNA counts in CPM for the forward (red) and reverse (blue) (bin = 100 bp) are plotted. Map of the genes (blue, forward; red, reverse). Genomic coordinates of the studied loci are indicated below. The different scale bars are indicated on the right. (**C**) Average 3D projection of the PAK chromosome at 0, 3, 7, and 13 min after addition of phage PAK_P3. The color of the structure is the same as in Fig. 1E.

We performed, in two replicates, HiC on a PAK_P3-infected exponential culture of the bacterial strain PAK at a multiplicity of infection (MOI) of 25 (fig. S3A). Given the fast PAK_P3 infection cycle (∼20 min), we sampled the culture at 0, 3, 5, 7, 10, 13, and 16 min postaddition of phage (T0 to T16), fixed the cells, and processed the pellets for HiC. Computation of relative copy number of each chromosome (phage and bacteria) during the time course showed a rapid increase in the number of phage DNA molecules from 0 to 3 min, a relative stabilization between 3 and 7 min, followed by a new increase between 7 and 13 min (fig. S3B). These results were in line with the expected infection cycle of the PAK_P3 phage, with an entry of the phage within the cell population (0 to 3 min), hijacking of the host (3 to 7 min) before the replication of the phage genome and its encapsidation (7 to 16 min) (*27*). A comparison of HiC maps recovered from cells along the kinetics showed a progressive decondensation of the host chromosome, accompanied by a disappearance of TIDs and cis contacts (Fig. 2, A and B, and fig. S3C) (*16*). This phenomenon was observed at 13 min postinfection, where the entire main diagonal appears affected compared to the noninfected culture (Fig. 2, A and B). We also observed a loss of local borders, as well as of other 3D structures such as stripes and loops (Fig. 2B). In contrast, the secondary diagonal signal was retained, suggesting the persistence of replichore bridging (Fig. 2A and figs. S3C and S4) and that the SMC-ScpAB complex is not affected by the infection at this stage. Incidentally, the average 3D structure of the PAK genome during the infection reflected the structural changes inferred from the HiC map: a conservation of the global organization accompanied by a disruption of local folding of the chromosome (Fig. 2C). These observations, reminiscent of the effect of rifampicin on bacterial chromosome architecture (*14*, *16*), agreed with a progressive arrest of the host transcription concomitant to the hijacking of the transcriptional machinery by phage PAK_P3 (*27*).

### PAK_P3 genome rapidly decondenses and reorganizes following its transcriptional program

**Fig. 3.**
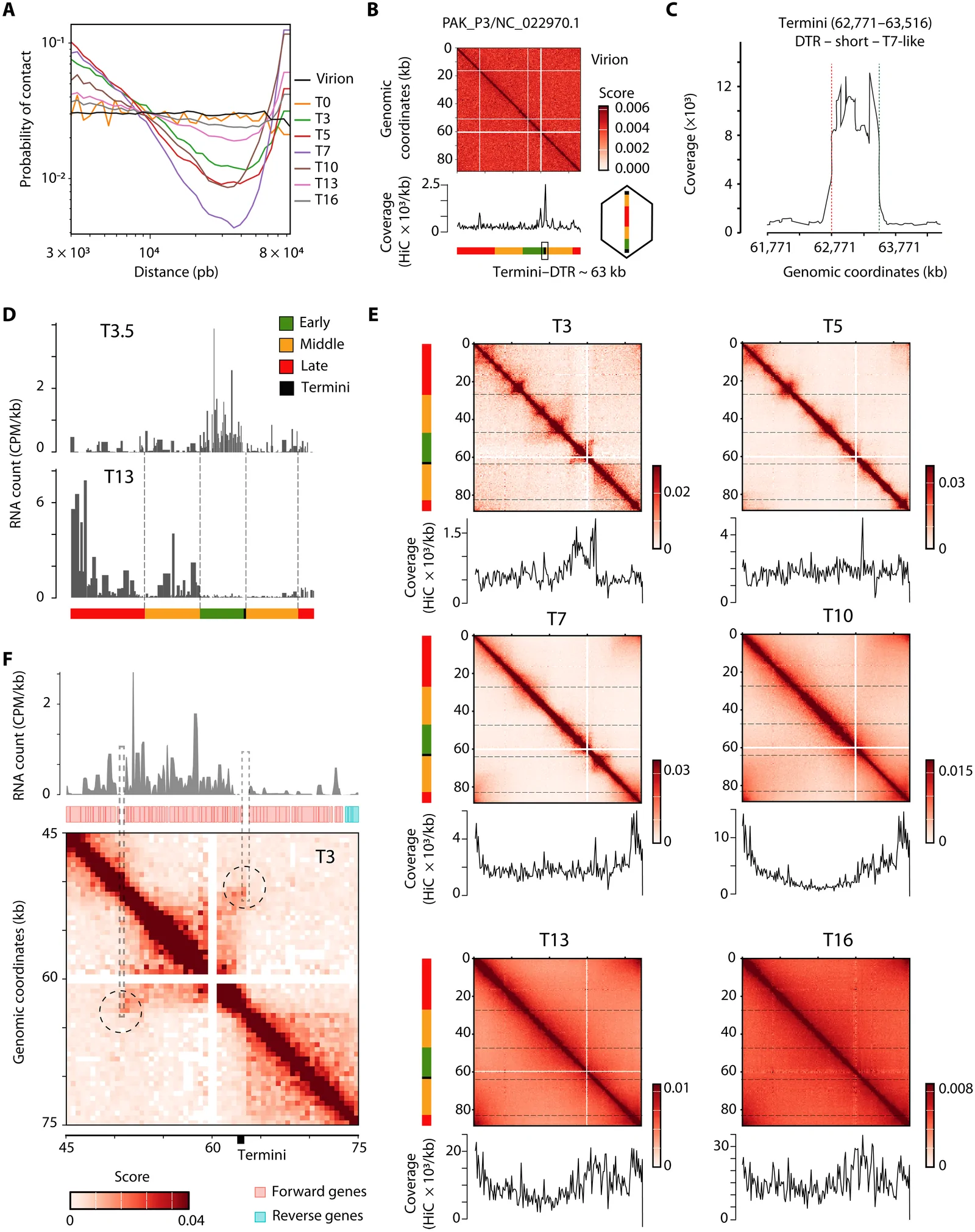
Dynamics of phage PAK_P3 genome during infection. (**A**) Normalized contact in function of the genomic distance for the PAK_P3 genome at the different time points [*p*(*s*)]. Normalized scores and genomic coordinates are in log scale. The color of each time point is indicated on the right (black line represent the phage within its capsid). (**B**) Contact map of PAK_P3 (bin = 500 bp) within its capsid. HiC coverage is indicated below the contact map. A linear colored scheme (red, orange, green, and black) is drawn below coverage plot and refers to the transcriptional program of the phage and the location of the termini (D). A scheme of the phage within its capsid is indicated on the right. (**C**) PhageTerm output on the localization and the profile of the termini. (**D**) RNA count for T3.5 and T13 from previous publication (*27*). Colored segments below the plot indicate the different gene clusters corresponding to early (green), middle (orange), and late (red) transcribed genes. Termini determined by PhageTerm are indicated (black). Dashed lines correspond to the borders of the early, mid, and late clusters, respectively. (**E**) Contact maps of PAK_P3 at time points 3, 5, 7, 10, 13, and 16 min. The HiC coverage is plotted below each map (bin = 1 kb). (**F**) Magnification of the contact map at the locus encompassing the loose looping structure (45 to 75 kb). RNA count (*T* = 3.5 min) is plotted on the right of the map. Forward (red) and reverse (blue) genes as well as termini (black box) are also indicated. Dashed area points to the two anchor loci positioned at the tip of the structure (dashed circles).

In addition to providing information about the host’s genome organization, HiC also revealed the dynamic changes experienced by the PAK_P3 genome during the infection time course. We then analyzed the architecture of the phage genome for each time point, from T0, when the phage is still inside its capsid, until T16 at the end of the infection (Fig. 3 and fig. S5A). At T0, the contact decay curve or genomic distance law *p*(*s*) was nearly flat, suggesting relatively equal contacts between pairs of DNA segments independently of the distance separating them (Fig. 3A, orange curve). This is consistent with a highly condensed, constrained, and organized genome where DNA is tightly packed within the capsid, as already described (*34*). To confirm this result, we applied HiC directly on a suspension of viral particles and obtained a *p*(*s*) curve and a contact map exhibiting the same features (Fig. 3, A and B, and fig. S5). We then used PhageTerm software (*35*) that predicts DTR (Direct Terminal Repeats) termini (T7-like) located around 63 kb (Fig. 3, B and C). Our results confirmed the linear topology of the phage within its capsid and the absence of circular permutation of its genome (Fig. 3B). The absence of a recurrent pattern in the contact map also suggests that, even if PAK_P3 genome is highly condensed in the capsid, it does not adopt always the same configuration. In contrast, the *p*(*s*) curve transitions from flat to sharply declining during the first time points demonstrated a rapid decondensation of the viral chromosome as it entered the host cytoplasm, with a minimum reached between 5 and 10 min (Fig. 3A and fig. S5). Then, the *p*(*s*) gradually switched back toward a flat state, and, at 16 min, it had nearly returned to its initial T0 state, suggesting that most of the newly synthesized PAK_P3 genomes were encapsidated into formed viral particles. Comparison between transcriptional profiles (*27*) and contact maps generated over the course of infection further highlighted the dynamic structural changes experienced by the phage genome (Fig. 3, D and E). Segments of the genome indeed showed an increase in medium-range contacts that correlated with regions carrying genes expressed at early, middle, and late stages of infection (Fig. 3, D and E). As these domains do not correlate with specific GC content, a potential bias of HiC experiments, it is likely that the structures observed with HiC were linked to transcription of the phage genome (fig. S6). In addition, at 3 min and in both replicates, a 14-kb region encompassing part of the early expressed genes adopted a loose looping structure and isolated itself from the rest of the genome (Fig. 3, E and F, and fig. S6). This discrete structure, which encompasses on one side the termini of the phage, points to the possible action of one or more specific factors involved in structuring phage DNA. The tip of the structure consisted of two sequences devoid of genes and transcription (Fig. 3F, dashed circles point to the tip of the structure and dashed areas to the anchor sequences). We also observed the apparition of a circular signal along the phage cycle with a clear increase between 3 and 10 min, followed by a decrease at 13 min [Fig. 3, A and D, increase in the *p*(*s*) curve at the end of the genome and increase in the HiC signal in the corner of the contact map]. This observation implies a circularization of the phage genome presumably by recombination of the DTR termini as already described (*35*). These observations also point to two potential types of replication of the phage DNA previously observed for Myoviridae (*36*): a rolling circle replication that generates concatenates of phage genomes or a theta replication that solely produces circular molecules. Analysis of the HiC coverage showed a clear skew from both extremities to the center between 7 and 10 min supporting the existence of circular molecules and the theta replication hypothesis associated with a single start site for replication (Fig. 3D). Nonetheless, this bidirectional replication could also be coupled to a rolling circle replication as already described for phage lamba (*37*).

Together, our data showed that the temporal regulation of PAK_P3 genome structure is strongly associated with the carefully regulated activation of its gene expression program, starting at the earliest stages of infection (3 min). Our results also strongly suggest that phage genomes, such as cellular organisms, are actively structured.

### PAK_P3 exhibits a specific interaction pattern with the host chromosome

**Fig. 4.**
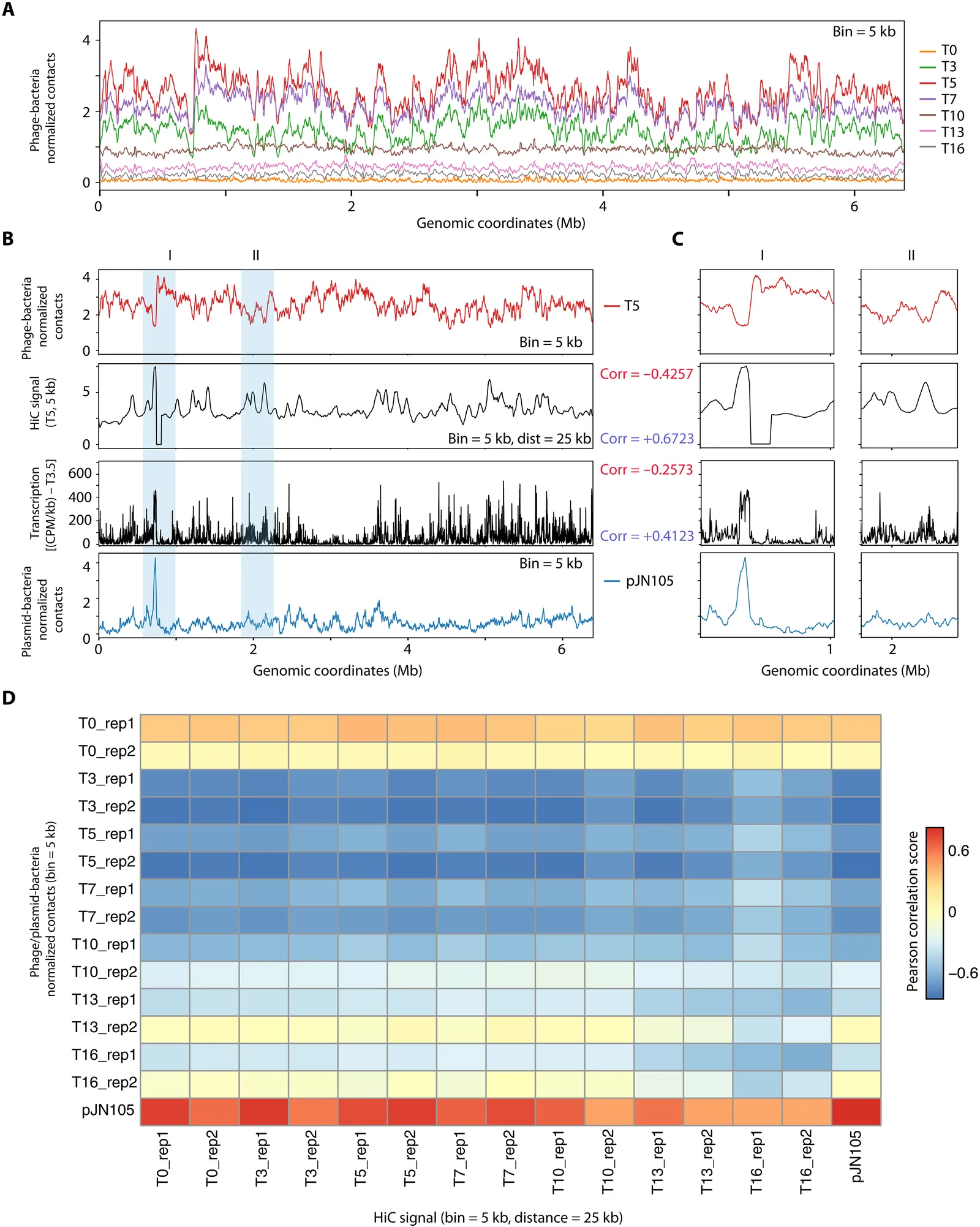
Interactions between PAK_P3 and *P. aeruginosa* chromosome. (**A**) 4C-like contact of PAK_P3 genome with the PAK genome (5-kb bins). Colors are the same as for Fig. 3 and are indicated on the right of the graph. (**B** and **C**) From top to bottom: 4C-like contact (5-kb bins) of PAK_P3 genome with PAK genome after 5 min of infection, HiC signal at short range (distance = 25 kb and bin = 5 kb), transcription signal of the PAK genome at 3.5 min in CPM per kilobase, and 4C-like contact (5-kb bins) of plasmid pJN105 with the PAK genome. Genomic coordinates of the PAK genome are indicated at the bottom of the plots. Pearson correlation coefficients between 4C-like contact profiles (red, PAK_P3 at T5; blue, pJN105) and either HiC or RNA signal is given on the right of the panel. Two magnifications of the profiles are plotted in (C) [areas I and II are indicated in blue in (B)]. (**D**) Matrix indicating the Pearson correlation score between the 4C-like contact of PAK_P3 or pJN105 genome with the PAK genome and the HiC signal at short range for the same time points (distance = 25 kb and bin = 5 kb) at the different time points.

We next computed the contacts between the phage DNA with the host chromosome at each time point during phage infection (Fig. 4A and fig. S7). The normalized score of contacts between PAK_P3 DNA molecules and the host chromosome increased from 0 to 7 min before decreasing until 16 min, in agreement with the packaging of the phage between 7 and 16 min isolating its DNA from the host. We also detected specific, enriched contacts of the phage with the host’s chromosome (Fig. 4, A and B; examples of valley and peak of interactions are labeled as areas I and II). These contacts suggested a specific underlying mechanism that may favor hijacking of the host transcriptional machinery. To test whether these contacts corresponded to specific localization of the phage genome and/or a more generic increased accessibility of the host nucleoprotein filament by episomes, we performed a HiC experiment on the *P. aeruginosa* strain PAK carrying the multicopy 6-kb plasmid pJN105. The 4C-like contacts plot (one vs. all) between the plasmid and the bacterial chromosome displayed a pattern inversely correlated with the phage contacts plot (Fig. 4, B and C; Pearson correlation score = −0.356), demonstrating different mechanisms of interaction of these two genomic entities with the host DNA and, therefore, different localizations within the cellular space. We then asked whether these different patterns could be linked to the HiC signal at short range or to host transcription activity, two signals known to be correlated (Fig. 1A). A plot of the contacts between PAK_P3 and *P. aeruginosa* chromosome after 5 min of infection regarding HiC signal at the same time or RNA profile at 3.5 min of infection hinted that PAK_P3 interacts preferentially with weakly transcribed regions of the chromosome (Pearson correlation score = −0.4257 and −0.2573, respectively; Fig. 4, B and C). On the contrary, the pJN105 4C-like contact plot exhibits a clear correlation with these two signals (Pearson correlation score = +0.6723 and +0.4123, respectively; Fig. 4, B and C). To go further in this analysis, we computed the Pearson correlation score between contact score and HiC signal as well as transcriptomic data for all time points and showed that, in contrast to plasmid pJN105, phage genome contacts during the first half of the infection cycle show an anticorrelation with HiC signal and transcriptional profile of the host chromosome (Fig. 4D and fig. S7).

Overall, the HiC temporal analysis of the infection cycle of phage PAK_P3 revealed an unexpected behavior of its genome that interacts preferentially with low active host chromosome loci, unlike a high-copy plasmid. Whether such a profile is due to an active positioning of the phage genome or is an indirect consequence of either the phage or the host metabolism remains to be elucidated.

## DISCUSSION

**Fig. 5.**
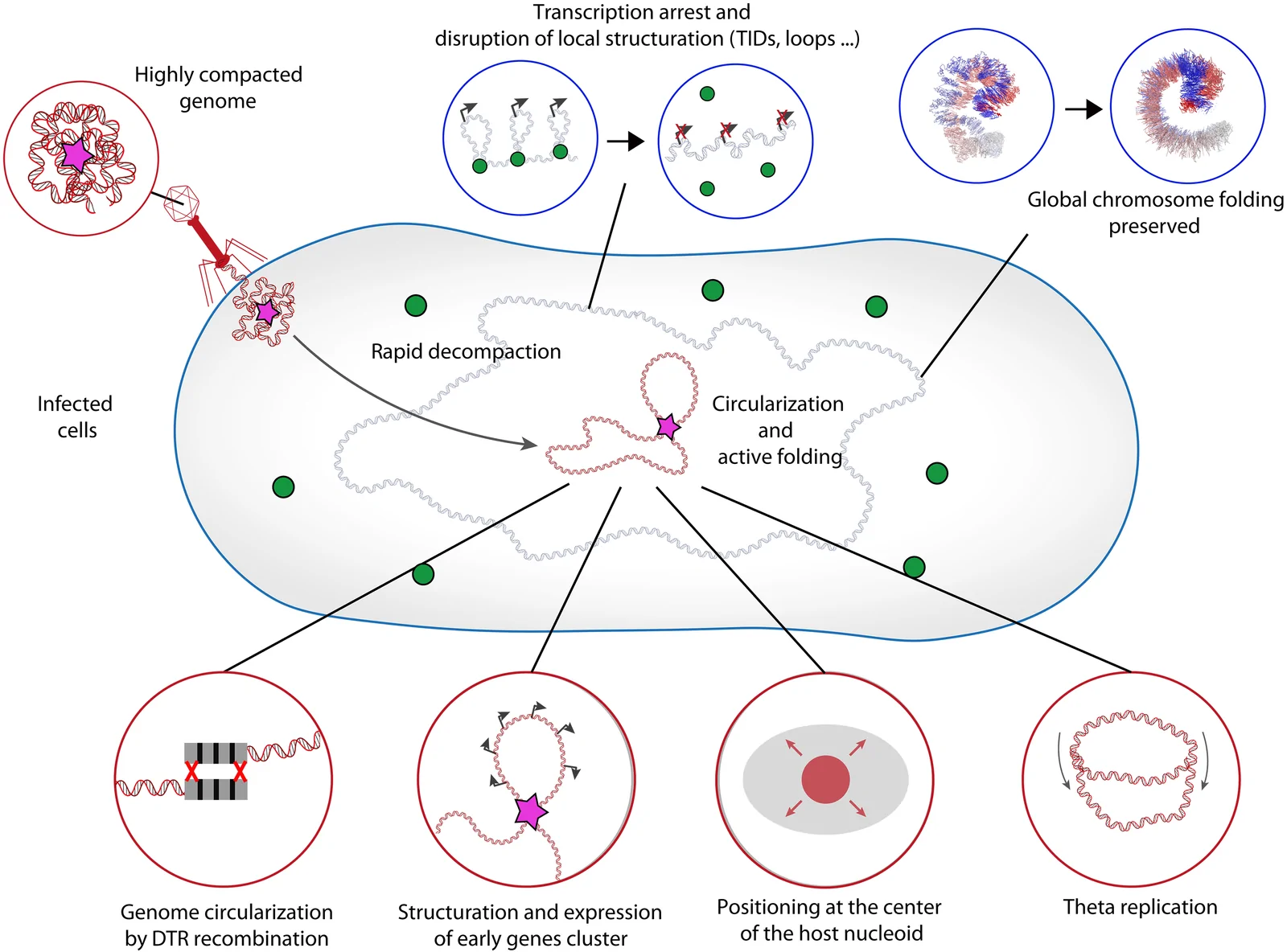
Model of PAK_P3 genome dynamics and impact on host chromosome during its infection cycle in *P. aeruginosa*. Red and blue DNA molecules correspond to phage and host genomes, respectively. Green circles refer to host proteins, while pink star refers to NAP-like factor present in the viral particles and responsible for the early structuration of the phage genome and/or genome circularization through DTR recombination. Black arrows correspond to transcription.

Since the discovery of phages more than a century ago, research on these highly diverse viruses has yielded fundamental insights into a broad range of processes related to DNA metabolism (*2*). Several studies have begun to decipher the mechanism of action of bacterial infection by phages, notably in *P. aeruginosa* (*24*, *26*, *27*, *38*). In the present work, we have deciphered the infection cycle of the virulent *Pseudomonas* phage PAK_P3 using proximity ligation–based methods. This pioneering work shed light on new processes concerning its genome organization, its dynamics, and its impact on the architecture of the *P. aeruginosa* chromosome and offered valuable insights into the complex interplay between this phage and its bacterial host (Fig. 5).

First, PAK_P3 infection led to a rapid decondensation of the host chromosome, loss of local bundle correlations with transcription, and alterations in chromatin structures. These observations are reminiscent of the effects of rifampicin on bacterial chromosome architecture (*14*, *15*) and confirm a gradual arrest of host transcription due to phage hijacking of the transcriptional machinery as previously demonstrated using transcriptomic data (*27*).

The temporal analysis of phage infection reported also provides critical insights into the dynamics of phage genomes during infection. The rapid decondensation of its genome upon entry into the host cytoplasm, followed by a recompaction phase, demonstrates dynamic changes in its structure during infection. This dynamic genome restructuring notably led to the formation of structural domains whose borders correlate with the phage transcriptional program. One notable observation comes from the early time point (T3) where the locus encompassing early expressed genes adopts a loose looping structure (Figs. 3 and 5). This result points to an active process that raises the question of the existence of NAP (nucleoid-associated protein)–like factors, noncoding RNAs, loop extrusion mechanisms, or plectoneme formation that could actively and rapidly structure the phage genome. Overall, our results demonstrate that phage genomes, similar to the genomes of cellular organisms, can be highly structured during their reproductive cycle. This could imply that structural factors may be encapsidated with the phage genome (Fig. 5, pink star). Among the peptides identified from the proteomic analysis of PAK_P3 virions (*39*), we have detected the factor Gp160 (YP_008857795.1), a small and basic protein (isoelectric point = 9.36) that is predicted to belong to the family of DNA binding protein, typical features of known bacterial NAPs (table S1). Moreover, this factor is encoded by a monocistronic gene, and its expression is not temporally regulated during the infection, suggesting that its expression is not mandatory during infection cycle (*27*). To strengthen the hypothesis regarding the involvement of specific phage proteins in the early structuration of the phage genome, additional studies using phage mutants and/or chromatin immunoprecipitation sequencing experiments could be engaged.

Our study also reveals that PAK_P3 phage interacts preferentially with specific locations of the *P. aeruginosa* genome. The fact that PAK_P3 and the multicopy plasmid pJN105 exhibit opposite interaction patterns demonstrates that MGEs have different ways of taking advantage of the host genome architecture. In the case of PAK_P3, our data show that it interacts with low-transcriptionally active areas. Some studies on bacteria have shown that active chromatin tends to localize at the periphery of the nucleoid (*16*, *40*). Therefore, our results suggest that PAK_P3 locates its genome at the center of the nucleoid, a location that may be favorable for hijacking the cellular machinery of *P. aeruginosa*, having access to mandatory metabolites or escaping the cell defense mechanisms. The development of dedicated tools for imaging PAK_P3 genome in infected cells would be required to provide additional evidence supporting the above hypothesis.

In summary, our study highlights the infection strategy used by a virulent phage infecting *P. aeruginosa* and uncovers intricate interactions between viral and host genomes. These findings open avenues for further exploration into the mechanisms underlying phage infection and their impact on host chromosome architecture. Understanding these processes at a molecular level will provide an advanced comprehension of virus-host interactions and may open avenues for developing phage-based therapies.

## MATERIALS AND METHODS

### Experimental design

The objective of our study is to decipher the choreography of PAK_P3 genome and its interplay with host chromosome during infection of *P. aeruginosa*.

### Bacteriophage and bacterial strains

Strains (PAK and PAK + pJN105) were grown in Lennox broth (LB) or on Lennox agar at 37°C. PAK_P3 phage was amplified in exponential growing cultures of strain PAK for ∼6 hours. Bacterial debris was removed first by centrifugation at 5200*g* for 10 min at 4°C, and then cell lysate supernatants were filtered through a 0.22-μm filter and kept at 4°C. Phage stock concentrations were then quantified by spot dilution assay (*39*).

### Kinetics of phage infection

Strain PAK was grown until mid-exponential phase (optical density = 0.3) in 80 ml of LB. PAK_P3 phage was then added at a MOI of 25 (*27*). Eight milliliters of aliquots of the phage bacterial culture were removed at specific intervals of the phage bacterial infection cycle: 0, 3, 5, 7, 10, 13, 16 min. One milliliter was used to perform a spot assay and quantify phage loads at the different intervals of infection. The remaining 7 ml was immediately transferred into 15-ml falcon tubes containing 1 ml of 35.5 to 37% formaldehyde to perform HiC experiments. Tubes were then incubated for 30 min under shaking at room temperature, followed by incubation at 4°C for another 30 min. Formaldehyde was quenched by adding 2 ml of 2.5 M glycine, followed by an incubation of 20 min under shaking. Pellets were recovered by a centrifugation of at 10,000*g* for 10 min at 4°C, washed in phosphate-buffered saline, and then recentrifuged using the same settings. Pellets were then frozen in dry ice and stored at −80°C until use.

### HiC libraries

Proximity-ligation libraries were generated using the Arima kit (Arima Genome-Wide HiC Kit). Samples were first incubated for 1 hour on ice and then resuspended in 1 ml of sterile water and transferred to 2-ml Precellys tubes containing glass beads of 0.1 and 0.5 mm in diameter (Precellys, Bertin Technology). Cells were disrupted using the Precellys apparatus (Precellys Evolution) and the following program (7500 rpm, 6 cycles 30-s on/30-s off, 4°C). Tubes were then centrifuged at 1000*g* for 1 min, and 700 μl of lysate was recovered and transferred to a new 1.5-ml Eppendorf tube. Tubes were centrifuged at 16,000*g* for 20 min at 4°C. Supernatant was carefully removed, and pellet was resuspended in 45 μl of water. We then followed the Arima protocol with the addition of a centrifugation step after the digestion (yellow solution) as previously described (*41*). Briefly, after the digestion step, tubes were centrifuged at 16,000*g* for 20 min at 4°C. Supernatant was carefully removed, and pellet was resuspended in 100 μl of water before applying the next step of the kit (blue solution) and following the protocol to the end. DNA was then purified using AMPure beads and eluted in 130 μl of water before processing for sequencing.

### Libraries processing and sequencing

HiC genomic libraries were first sheared at a mean size of 500 bp using a Covaris S200 apparatus and then processed on streptavidin beads using the Colibri kit (Thermo Fisher Scientific) as previously described (*42*). Libraries were sequenced on NextSeq or NovaSeq apparatus.

### HiC data analysis

Contact maps were generated using hicstuff (*43*) and predigested reads (bowtie2, very sensitive local mode, mapping quality of 30) and a reference FastA files containing the phage PAK_P3 (or the plasmid pJN105) and the PAK genome. Contact maps were then binned at different resolutions (500 bp, 1 kb, 2 kb, and 5 kb), balanced using cooler (*44*), analyzed, and displayed using different dedicated R packages (HiContacts, GenomicRnages, InteractionSet, and HiCexperiments) (*45*). All the scripts for processing the data are available at the following link: https://github.com/mmarbout/PAK_P3_infection_analysis.

### RNA sequencing analysis

RNA raw fastq files (*27*) were recovered from Sequence Reads Archive (SRA) database and processed using homemade script (tinymapper, https://github.com/js2264/tinyMapper). Processed RNA files for phage transcription signal were also recovered from the corresponding study [National Center for Biotechnology Information (NCBI) Gene Expression Omnibus (GEO) portal: GSE76513). RNA data were combined with HiC using different R packages and plotted using R.

### 3D projection

Mcool files were used as input for the workflow 3D genome builder (https://github.com/data-fun/3d-genome-builder). Protein Data Bank (PDB) files obtained were then modified using custom script and visualized using PyMOL.

### Prediction of NAPs

Using HMMsearch (hmmer.org), we searched for PAK_P3 proteins homologous to 14 known bacterial NAPs and histones (HU, H-NS, Lsr2, HMF, Hc1, Fis, IHF-A, IHF-B, Dps, Hfp, MvaT, CbpA, XrvA, and the bacterial histones) but detected no statistically significant matches. We next screened PAK_P3 proteins with HMMsearch against Pfam-A (release 35.0) and selected domains annotated in pfam2go (version 2025/04/29) (*46*) as “DNA binding” or “transcription factor” domains. Only YP_008857685.1 (gp50), containing a predicted DNA_pol_A domain, was identified. Last, on the basis of NAP criteria (*47*), proteins from phage PAK_P3 were screened for short length (≤300 amino acids), predicted DNA binding activity, and monocistronic gene organization. Size-filtered proteins were first analyzed using DNA Binder (*48*), followed by Operon Mapper (*49*). Ten proteins were retained and subsequently examined for their presence in PAK_P3 virions (*39*) (table S2).

### Statistics and reproducibility

No data were excluded from the analyses. Statistical tests state in the manuscript were done using R and the ad hoc function.
