# Supplementary material for: Bacteriophage PAK_P3 genome structuration and dynamics during infection of Pseudomonas aeruginosa reveal specific interaction patterns
Source: Sci Adv. 2026 Aug 26;12(35):eaef2512. doi: 10.1126/sciadv.aef2512 (PMC13510664; doi:10.1126/sciadv.aef2512)
Supplement: Supplementary file 1 [file sciadv.aef2512_sm.pdf]

Science Advances
AAAS

Supplementary Materials for

Bacteriophage PAK_P3 genome structuration and dynamics during infection of *Pseudomonas aeruginosa* reveal specific interaction patterns

Amaury Bignaud *et al.*

Corresponding author: Martial Marbouty, martial.marbouty@pasteur.fr

Sci. Adv. **12**, eaef2512 (2026)
DOI: 10.1126/sciadv.aef2512

This PDF file includes:

Figs. S1 to S7
Tables S1 and S2

Fig. S1. **Contact map at various resolutions and of the replicates.**

(**A**) From left to right: 1 kb-resolution, 2 kb-resolution and 5 kb-resolution Hi-C contact maps of the strain PAK. Genomic coordinates are indicated on the left and on the top of the contact maps while scale bars on the right. (**B**) 5 kb-resolution Hi-C contact maps of the two replicates for the strain PAK. (**C**) P(s) contact law for the two replicates.

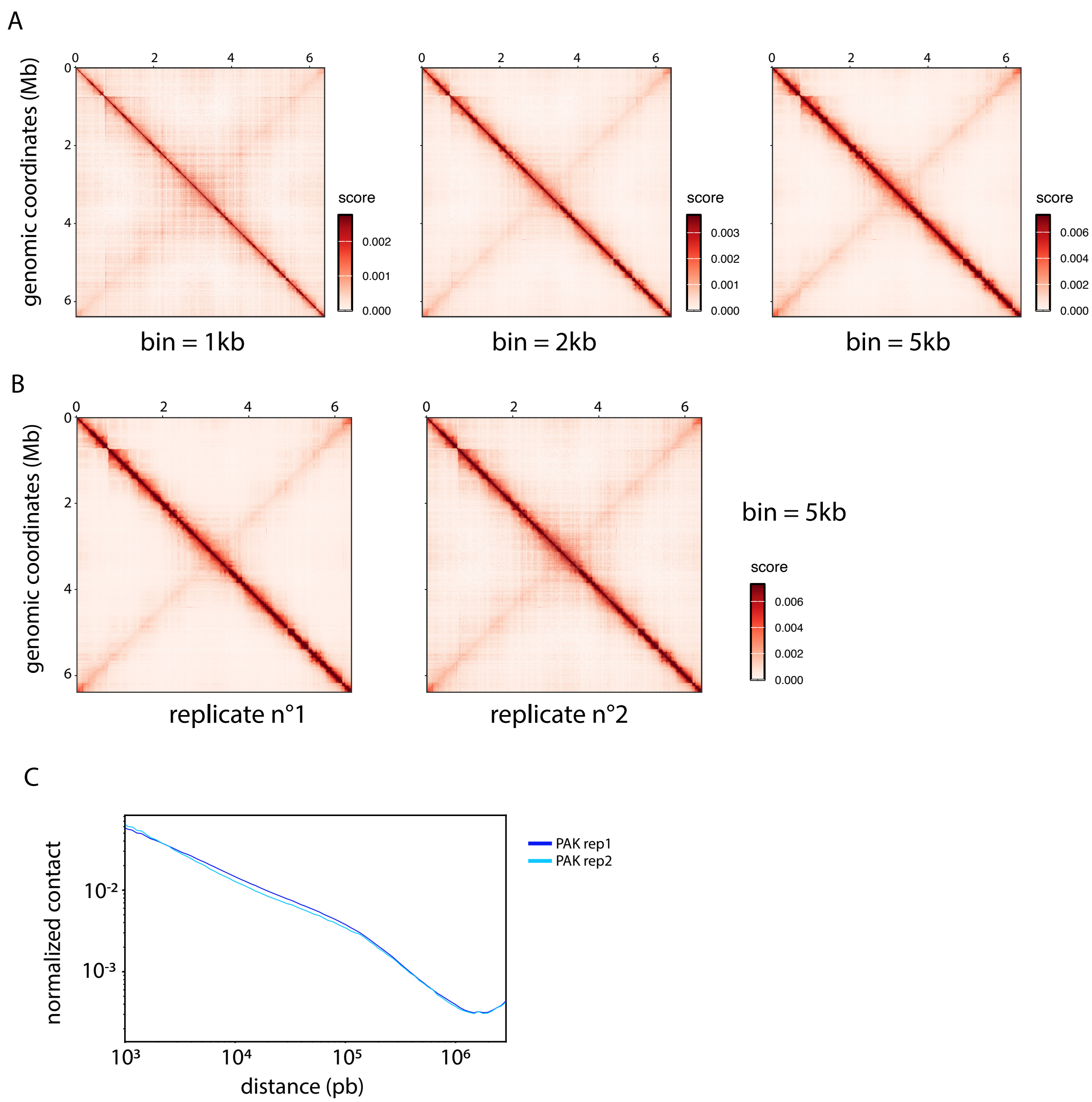

Fig. S2. Compartments of *P. aeruginosa*.

From top to bottom: contact map of P. aeruginosa strain PAK (bin = 5kb). Value of the five first eigen vector (EV1 to EV5).

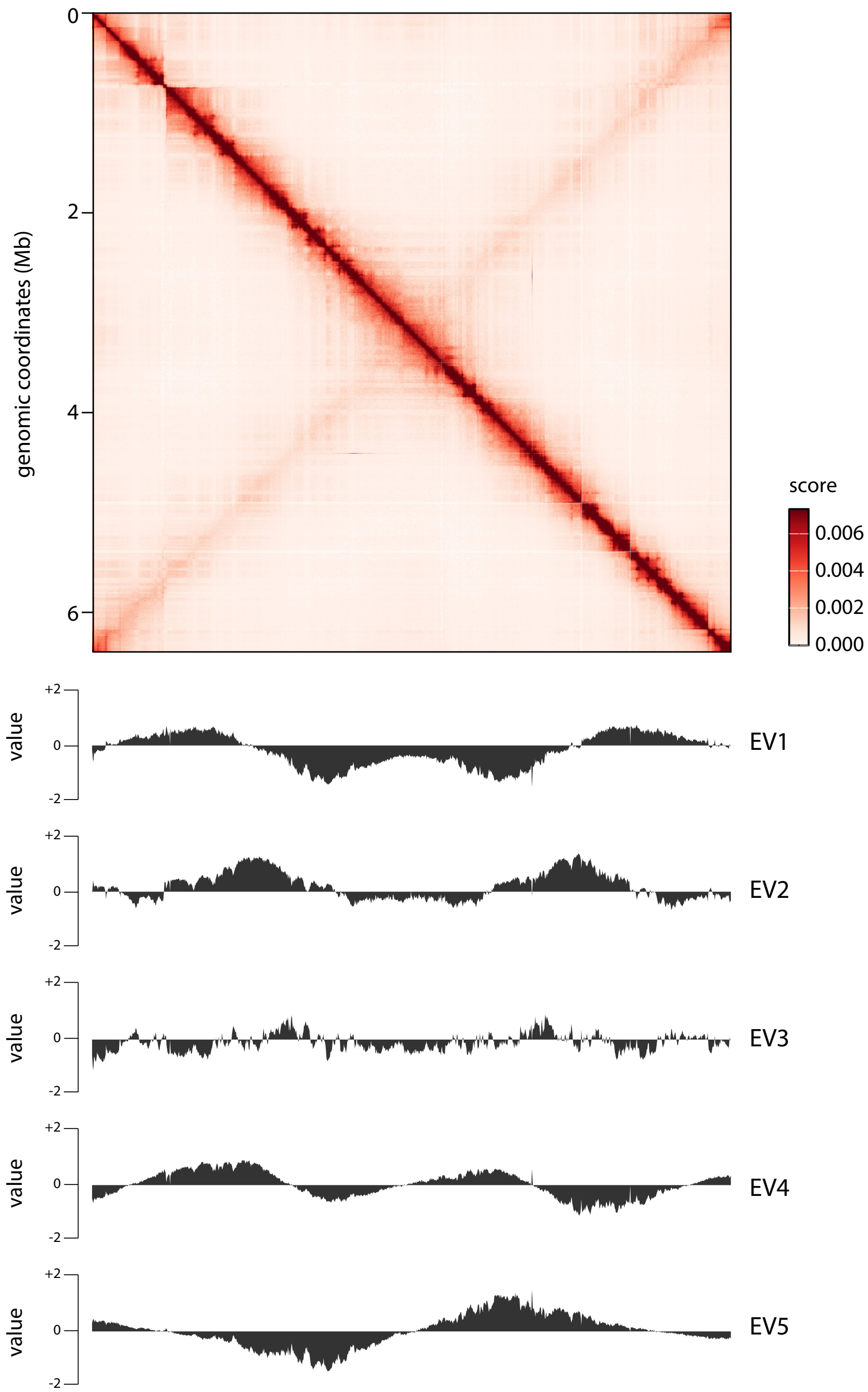

Fig. S3. Replicates of PAK_P3 infection cycle.

(**A**) Scheme of the experimental procedure. (**B**) Graphic of PAK (blue) and PAK_P3 (red) genomes abundances in function of time. (**C**) 5 kb-resolution ratio contact maps of the strain PAK during the PAK_P3 time course infection cycle. Each map is a comparison between a defined time point and the time 0 minute. Genomic coordinates are indicated on the map. A log scale bar is indicated on the right of the contact maps.

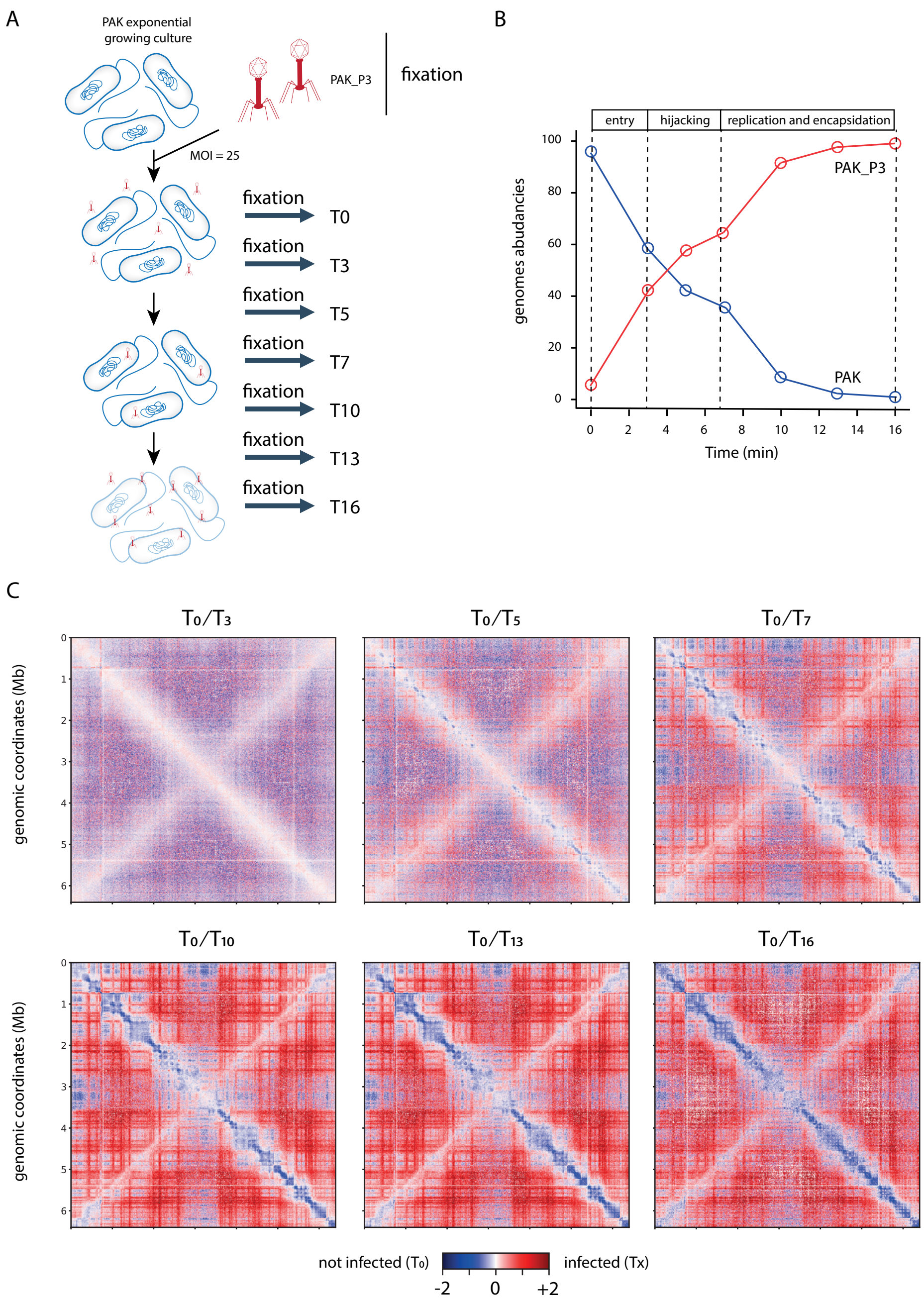

Fig. S4. Contact map of replicates of PAK during infection cycle.

5 kb-resolution contact maps of strain PAK for time points 0, 3, 5, 7, 10, 13 and 16 minutes (bin = 5kb). Genomic coordinates are indicated on the left of the maps. (**A**) Replicate 1. (**B**) Replicate 2.

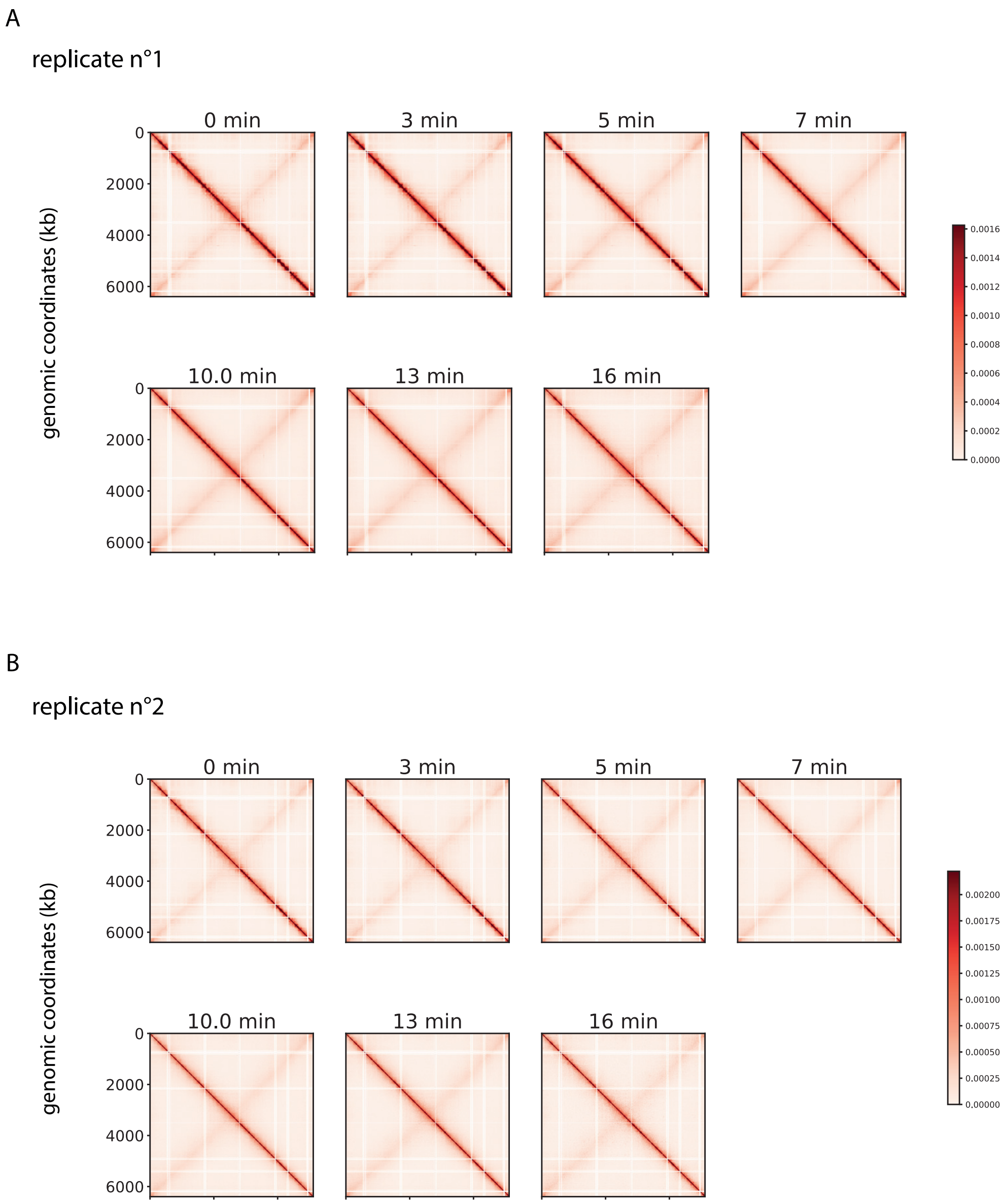

Fig. S5. Contact map of replicates of PAK_P3 during infection cycle.

(**A**) Normalized contact in function of the genomic distance for the PAK_P3 genome at the different time points and for the two replicates. Normalized scores and genomic coordinates are in log scale. The color of each time point is indicated on the right. (**B**) Contact maps of PAK_P3 for time points 0, 3, 5, 7, 10, 13 and 16 minutes (bin = 500 pb). Genomic coordinates are indicated on the left of the contact map. Top: replicate 1; bottom: replicate 2. Panels on the right corner indicate the GC content of the PAK_P3 genome (bin = 1kb).

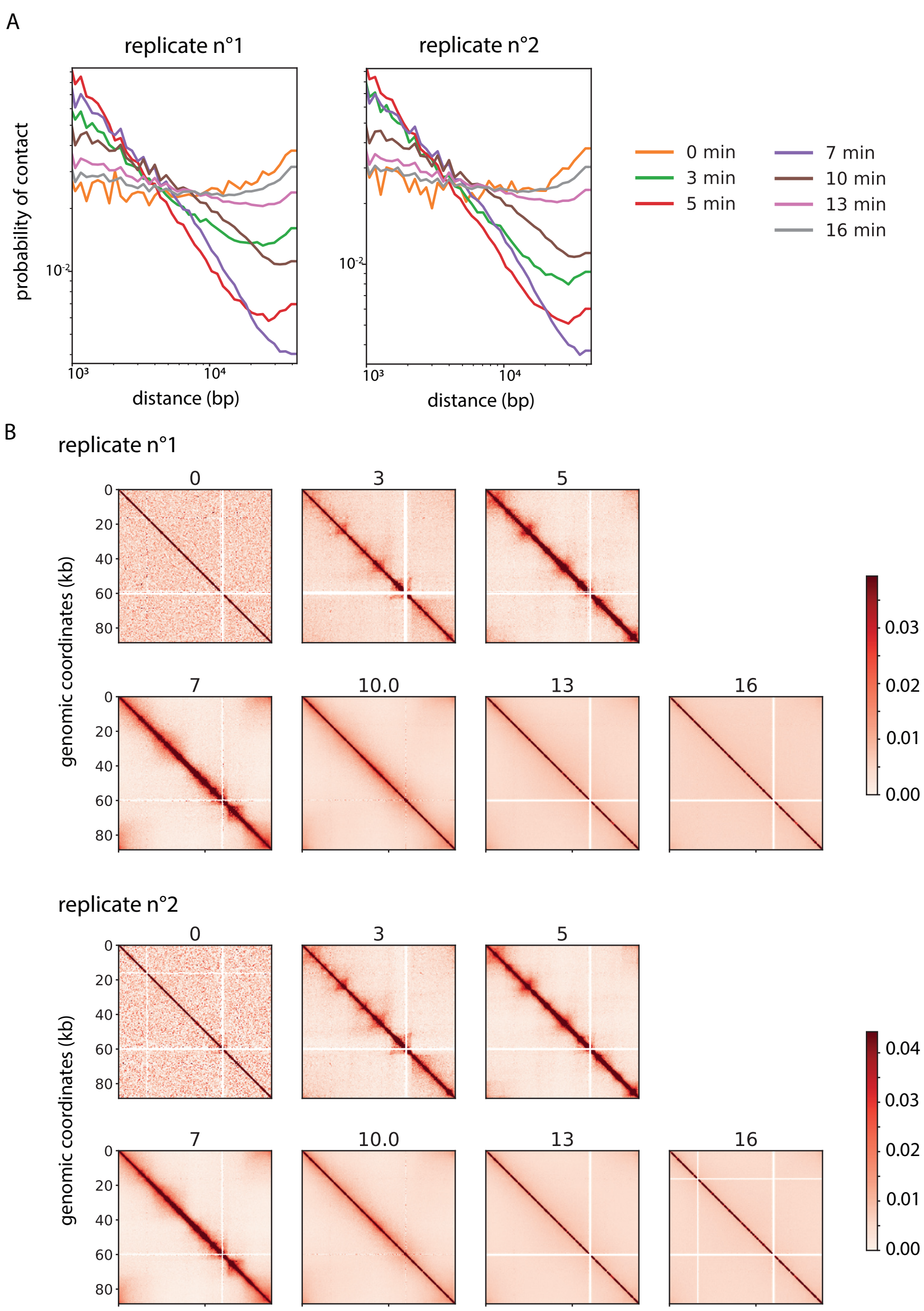

Fig. S6. Contact maps of PAK_P3 loosing-loop locus and GC content of the PAK_P3 genome.

(**A**) 500 pb-resolution of magnification of Hi-C contact maps of the PAK_P3 at 3, 5 and 7 minutes between 45 to 75 kb. Genomic coordinates are indicated on the map. Scale bar is indicated on the right. Top: replicate 1; bottom: replicate 2. (**B**) GC content along the PAK_P3 genome (bin = 1kb). Schemes of the cluster (early, middle, late), the genes (forward and reverse) and the termini (black) are indicated below the plot.

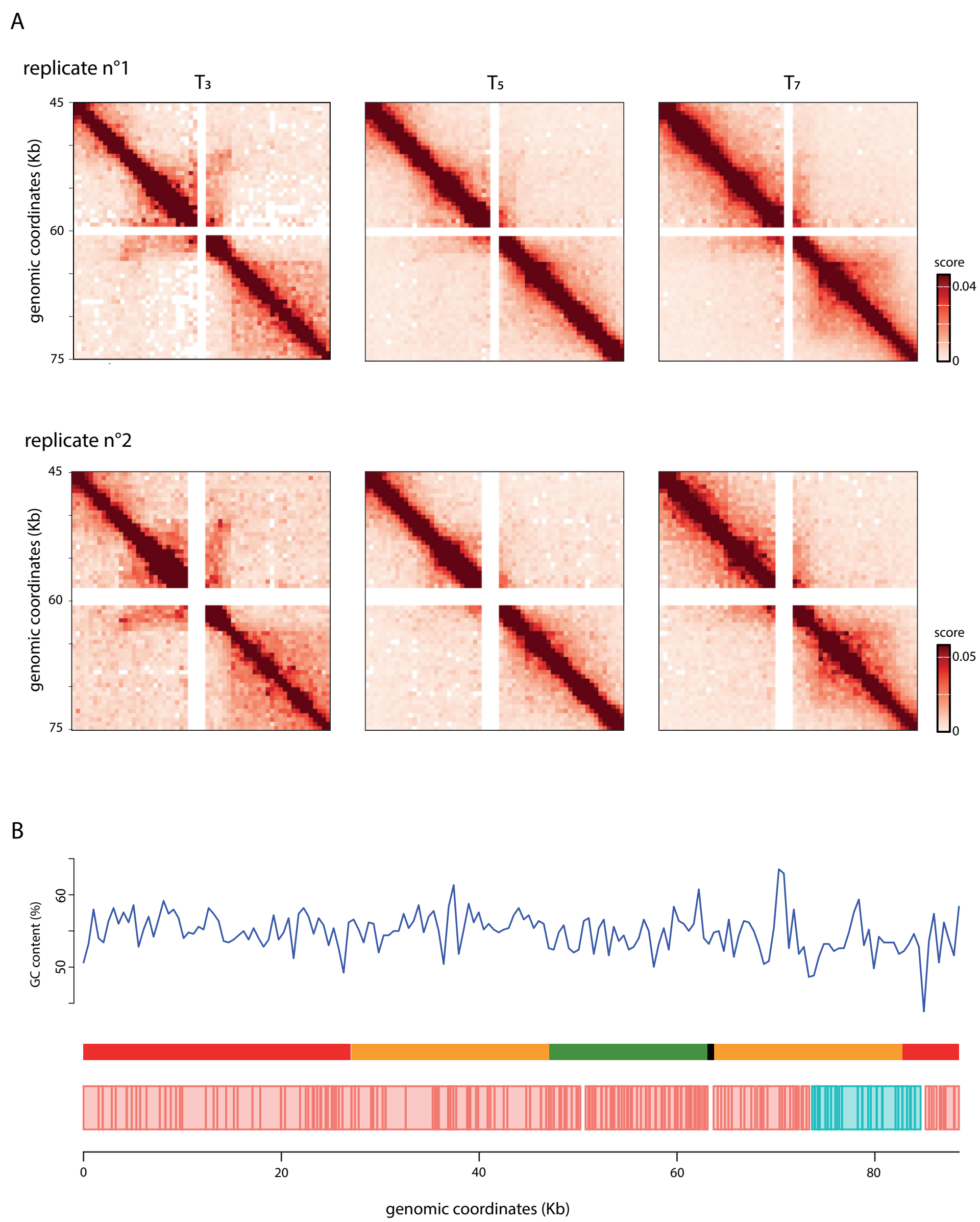

Fig. S7. Contact of PAK_P3 with PAK genome.

(**A, B**) 4C-like contact (10 kb bins) of PAK_P3 genome with the PAK genome at the different time points and for the two replicates (A - replicate 1; B - replicate 2). Genomic coordinates of the PAK genome are indicated below the graph. (**C**) matrix indicating the Pearson correlation score between the 4C-like contact of PAK_P3 genome or pJN105 with the PAK genome at the different time points (1 kb bin) and the transcriptomic signal at T0, T3.5 and T13.

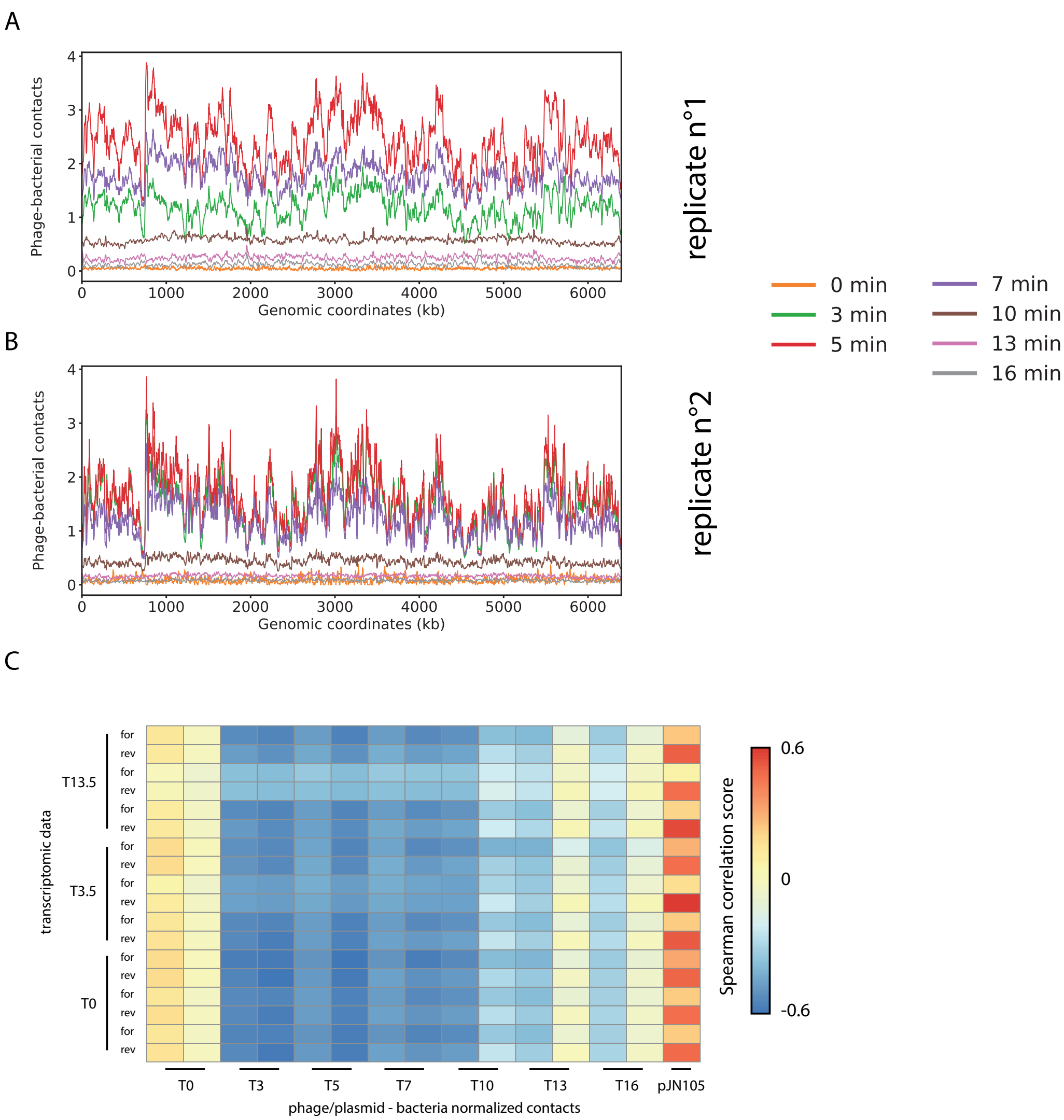

Table S1. NAPs candidate proteins encoded in PAK_P3 genome

**Protein ID**	**Protein name**	**Length** (aa)	**Predicted function (GFF)**	**presence in PAK_P3 particles ***
YP_008857687.1	gp52	61	Protein of unknown function	No
YP_008857715.1	gp80	70	Protein of unknown function	No
YP_008857729.1	gp94	78	Protein of unknown function	No
YP_008857733.1	gp98	81	Protein of unknown function	No
YP_008857735.1	gp100	171	Protein of unknown function	No
YP_008857741.1	gp106	152	Protein of unknown function	No
YP_008857772.1	gp137	76	Protein of unknown function	No
YP_008857795.1	gp160	58	Protein of unknown function	Yes
YP_008857798.1	gp163	100	Protein of unknown function	No
YP_008857799.1	gp164	272	Terminase large subunit	No

Table S2. Datasets used in the present study.

sample	id	library type	Bioproject	SRA accession
PAK_P3 infection (rep1) T0	MM37	HiC	PRJNA1331554	SRR35511902
PAK_P3 infection (rep1) T3	MM53	HiC	PRJNA1331554	SRR35511901
PAK_P3 infection (rep1) T5	MM54	HiC	PRJNA1331554	SRR35511895
PAK_P3 infection (rep1) T7	MM55	HiC	PRJNA1331554	SRR35511894
PAK_P3 infection (rep1) T10	MM56	HiC	PRJNA1331554	SRR35511893
PAK_P3 infection (rep1) T13	MM57	HiC	PRJNA1331554	SRR35511892
PAK_P3 infection (rep1) T16	MM58	HiC	PRJNA1331554	SRR35511891
PAK_P3 infection (rep2) T0	MM142	HiC	PRJNA1331554	SRR35511890
PAK_P3 infection (rep2) T3	MM143	HiC	PRJNA1331554	SRR35511889
PAK_P3 infection (rep2) T5	MM144	HiC	PRJNA1331554	SRR35511888
PAK_P3 infection (rep2) T7	MM145	HiC	PRJNA1331554	SRR35511900
PAK_P3 infection (rep2) T10	MM146	HiC	PRJNA1331554	SRR35511899
PAK_P3 infection (rep2) T13	MM147	HiC	PRJNA1331554	SRR35511898
PAK_P3 infection (rep2) T16	MM148	HiC	PRJNA1331554	SRR35511897
PAK + pJN105 expo	FG0169	HiC	PRJNA1331554	SRR35511896
PAK_P3 viral particles	MM239	HiC	PRJNA1331554	SRR38017185
PAK_P3 infection 0 min	T0_rep1	RNAseq	GSE76513 / PRJNA307603	SRX1516053
PAK_P3 infection 0 min	T0_rep2	RNAseq	GSE76513 / PRJNA307603	SRX1516054
PAK_P3 infection 0 min	T0_rep3	RNAseq	GSE76513 / PRJNA307603	SRX1516056
PAK_P3 infection 3.5 min	T3_rep1	RNAseq	GSE76513 / PRJNA307603	SRX1516047
PAK_P3 infection 3.5 min	T3_rep2	RNAseq	GSE76513 / PRJNA307603	SRX1516048
PAK_P3 infection 3.5 min	T3_rep3	RNAseq	GSE76513 / PRJNA307603	SRX1516049
PAK_P3 infection 13 min	T13_rep1	RNAseq	GSE76513 / PRJNA307603	SRX1516050
PAK_P3 infection 13 min	T13_rep2	RNAseq	GSE76513 / PRJNA307603	SRX1516051
PAK_P3 infection 13 min	T13_rep3	RNAseq	GSE76513 / PRJNA307603	SRX1516052
